# 16S rRNA Gene Amplicon Sequencing of Microbial Communities Involved in Anaerobic Bulking in a Mesophilic Expanded Granular Sludge Bed Reactor Treating Wastewater Discharged from a Japanese-Style Thickened Worcestershire Sauce-Producing Factory

**DOI:** 10.1128/MRA.00801-19

**Published:** 2019-09-05

**Authors:** Takeshi Yamada, Jun Harada, Yuki Okazaki, Tsuyoshi Yamaguchi, Atsushi Nakano

**Affiliations:** a Department of Applied Chemistry and Life Science, Toyohashi University of Technology, Toyohashi, Aichi, Japan; b Department of Civil and Environmental Engineering, National Institute of Technology, Matsue College, Matsue, Shimane, Japan; c Sumitomo Heavy Industries Environment Co., Ltd., Shinagawa, Tokyo, Japan; University of Southern California

## Abstract

We analyzed the prokaryotes in bulking and healthy sludge from a mesophilic expanded granular sludge bed reactor treating wastewater with high organic content by 16S rRNA gene amplicon sequencing. We tabulated the microbiota at the phylum level, providing a framework for avoiding sludge bulking.

## ANNOUNCEMENT

Expanded granular sludge bed (EGSB) reactors are the gold standard technology for industrial and agroindustrial wastewater treatment with high concentrations of organic matter (1). Despite their advantages, an issue of levitation/outflow of sludge (anaerobic bulking) has been recognized to cause changes in sludge sedimentation by overgrowth of filamentous microbes (2, 3). Information on microbiota associated with anaerobic bulking of an EGSB reactor for beverage wastewater has been reported (3); however, this information is not applicable to all EGSB reactors. To truly understand anaerobic bulking of EGSB reactors, information on anaerobic bulking-associated microbiota in EGSB reactors for treating various organic wastewaters should be collected.

Anaerobic bulking was observed in a full-scale mesophilic (30 to 33°C) EGSB reactor (established in Saitama in February 2014) treating wastewater discharged from a thickened Worcestershire sauce-producing factory at startup and during continuous operation. Using previously described measurement methods (2, 4, 5), we identified that the majority of the chemical oxygen demand (COD) in the wastewater supplied to the reactor comprised carbohydrates (3,840 mg COD/liter) and some organic acids (279 mg COD/liter). Two bulking sludges and two healthy granular sludges with different sampling times were collected from a sample port located 2 m from the bottom of the reactor (reactor volume, 60 m^3^) (Table 1). DNA extraction was performed using a previously described bead-beating method (4). The V4 region of the prokaryotic 16S rRNA gene was amplified using Blend *Taq* polymerase (Toyobo, Osaka, Japan) and 515F/806R primers (6). Amplification products with six replicates per sample were sequenced using the MiSeq instrument and MiSeq reagent kit v2 (300 cycles) (Illumina, San Diego, CA, USA) by Bioengineering Lab. Co., Ltd. (Kanagawa, Japan). The raw sequence reads were processed using the FASTX-Toolkit v0.0.13 (7) and Sickle v1.33 (8) to remove adaptor and primer sequences, ambiguous reads, low-quality sequences (with a quality score of ≤Q20), and reads of ≤40 bp. Quality-filtered sequences were merged using PEAR v0.9.10 with default settings (9), and merged sequences of ≤245 and ≥260 bp were removed using SeqKit v0.8.0 (10). 16S rRNA amplicon libraries for each sludge were clustered for assignment to operational taxonomic units (OTUs) using QIIME v1.9.1 (11) and the SILVA database (release 132) with 97% identity (12). Representative OTUs were tabulated at the phylum level; the results and indices of biodiversity, calculated using QIIME v1.9.1 (11), are displayed in Table 1.

**TABLE 1:** Summary of 16S rRNA gene amplicon profiles of the isolated microbiota

Analysis measure	Data by sample type (DRR accession no.)^*a*^			
	Bulking sludge A (DRR180051–DRR180056)	Bulking sludge B (DRR180045–DRR180050)	Healthy granular sludge A (DRR180039–DRR180044)	Healthy granular sludge B (DRR180033–DRR180038)
Sampling date	27 Feb 2014	7 Feb 2018	17 Dec 2014	27 Nov 2017
Diversity				
Estimated sample coverage	0.98	0.98	0.98	0.98
No. of OTUs	554.7	554.3	323.8	582.3
Shannon diversity	6.41	6.15	5.00	6.07
Simpson diversity	0.97	0.96	0.92	0.95
Chao1 estimator	837.8	947.1	531.3	932.9
ACE estimator^*b*^	845.2	887.4	555.6	924.7
High-quality reads	126,402	101,977	99,098	138,537
Relative abundance (%) of bacteria and archaeal phyla				
*Euryarchaeota*	36.2	10.5	17.1	15.2
*Acidobacteria*	0.1	ND	ND	0.1
*Actinobacteria*	0.1	0.1	ND	0.3
*Aegiribacteria*	0.6	ND	1.7	ND
*Armatimonadetes*	0.1	7.6	0.1	1.3
*Atribacteria*	0.5	0.1	0.2	0.2
*Bacteroidetes*	7.5	18.6	22.0	32.3
*Caldiserica*	ND	0.1	ND	ND
*Calditrichaeota*	0.2	ND	0.1	0.1
*Chloroflexi*	6.9	29.8	5.8	2.1
*Cloacimonetes*	0.3	0.4	ND	1.0
*Dependentiae*	0.1	ND	ND	1.7
*Edwardsbacteria*	ND	ND	0.5	ND
*Elusimicrobia*	0.1	0.1	ND	0.1
*Campylobacterota*	0.9	0.1	ND	ND
*Firmicutes*	3.4	11.8	1.0	29.0
*Kiritimatiellaeota*	0.7	1.6	ND	0.1
*Latescibacteria*	ND	ND	3.6	ND
*Lentisphaerae*	0.1	0.2	ND	0.1
*Mondulibacteria*	ND	0.5	7.8	ND
*Nitrospirae*	0.7	0.2	4.2	2.9
*Planctomycetes*	0.3	0.8	0.6	1.4
*Proteobacteria*	28.2	9.3	31.9	4.7
*Spirochaetes*	4.3	3.0	1.9	2.0
*Synergistetes*	1.8	1.2	ND	1.8
*Verrucomicrobia*	3.8	0.3	ND	0.2
*Zixibacteria*	ND	0.2	ND	0.3
Others	2.8	3.3	1.3	3.1

a ND, not detected.

b ACE, abundance-based coverage estimator.

In total, 101,977 to 126,402 and 99,098 to 138,537 high-quality reads were obtained from bulking and healthy granular sludge, respectively. The prokaryotic taxa were similar, but abundances differed among the four sludges. The major prokaryotes (abundance, >1%) common to all sludges were classified as *Euryarchaeota*, *Bacteroidetes*, *Chloroflexi*, *Firmicutes*, and *Proteobacteria* (Table 1). These data may provide insights into the prevention of anaerobic bulking in EGSB reactors by using suitable methods, such as setting the appropriate organic loading rate and adjusting the wastewater concentration and composition.

### Data availability.

The 16S rRNA gene amplicon data set was deposited in the NCBI Sequence Read Archive (SRA) under DRA accession number DRP005109 and SRA run accession numbers DRR180033 to DRR180056.
